# Quantifying Spatial Shadow Zones and Their Association With Hospital Falls in Acute Care Unit: Real-Time Location System Observational Study

**DOI:** 10.2196/75697

**Published:** 2025-09-10

**Authors:** Yen-Pin Chen, Yi-Chun Chen, Chen-Liang Lin, Chien-Yu Chi, Yi-Ying Chen, Bey-Jing Yang, Chien-Hua Huang

**Affiliations:** 1 Department of Emergency Medicine National Taiwan University Hospital Hsin-Chu Branch Hsinchu Taiwan; 2 Department of Emergency Medicine College of Medicine National Taiwan University Hospital Taipei Taiwan; 3 Guidercare Limited Taipei Taiwan; 4 Biomedical Technology and Device Research Labs Industrial Technology Research Institute Hsinchu Taiwan

**Keywords:** indoor positioning systems, IPS, patient safety, health care, ultra-wideband, UWB

## Abstract

**Background:**

Hospital falls represent a persistent and significant threat to safety within health care systems worldwide, impacting both patient well-being and the occupational health of health care staff. While patient falls are a primary concern, addressing fall risks for all individuals within the health care environment remains a key objective. Caregiver visibility and spatial monitoring are recognized as crucial considerations in mitigating fall-related incidents.

**Objective:**

This study aimed to investigate the association between the percentage of spatial shadow zone, defined as areas within an acute care unit unvisited by mobile workstations for prolonged periods, and the incidence of hospital falls and intensive care unit (ICU) transfers.

**Methods:**

This retrospective observational study was conducted in a 400-square-meter acute care unit of a tertiary hospital for over 210 days. An ultrawideband real-time location system was deployed to continuously track mobile workstations’ spatial coverage. Spatial shadow zones were defined as areas unvisited by mobile workstations for 60 continuous minutes. The primary outcome was hospital falls; the secondary outcome was ICU transfers. Multivariable logistic regression analysis, adjusted for patient-to-nurse ratio and day of week, was used to examine the association between the percentage of spatial shadow zone and these outcomes. Sensitivity analyses were performed by varying the spatial dilation distance (1-4 meters) and temporal shadow zone thresholds (15-90 minutes).

**Results:**

During this study’s period, 8 hospital falls and 89 ICU transfers occurred. Real-time location system validation indicated a mean positional error of 0.346 (SD 0.282) meters. In multivariable regression, a higher percentage of spatial shadow zone was significantly associated with an increased odds of hospital falls (odds ratio 1.02, 95% CI 1.01 to 1.03, *P*<.001). Conversely, a higher percentage of spatial shadow zone was associated with decreased odds of ICU transfer (odds ratio 0.99, 95% CI 0.99 to 0.99, *P*<.001). Sensitivity analyses demonstrated consistency of the association between spatial shadow zones and falls across varying parameter settings.

**Conclusions:**

This study provides novel evidence for a significant positive association between the percentage of spatial shadow zones and hospital falls, underscoring the critical role of caregiver visibility in fall prevention. The findings suggest that proactively minimizing spatial shadow zones through optimized hospital design, workflow strategies, and technology-enabled monitoring may be a valuable approach to enhance patient safety and reduce hospital falls in acute care settings.

## Introduction

### Background

Hospital falls constitute a persistent and significant threat to both patient safety and the occupational well-being of health care staff within global health care systems, an issue documented for decades [1-3]. Research indicates that the anticipated increase in hospital fall incidence, driven by the expanding global aging population [4,5], will likely exacerbate health care expenditures [6]. Mitigating hospital falls remains a paramount concern for health care institutions worldwide. Numerous factors contribute to the occurrence of these incidents, spanning patient-specific characteristics to environmental hazards and organizational protocols [4,7,8]. Within this multifaceted landscape, visibility, an extrinsic factor related to the physical environment and staff awareness, has emerged as a critical area of investigation.

Existing literature underscores the influence of unit layouts and spatial configurations on caregiver visibility and, consequently, patient safety. Some studies have focused on how ward design impacts nurses’ and other caregivers’ cognitive load in maintaining patient surveillance [9,10]. These works suggest that certain spatial arrangements may inadvertently create blind spots or areas with reduced visibility, potentially hindering timely intervention and increasing fall risk. Conversely, other research has explored the implementation of decentralized or satellite nursing stations situated closer to patient rooms as a strategy to enhance visibility and responsiveness [6,11]. Furthermore, the role of mobile workstations or nursing carts has been considered as a means to improve caregiver proximity and visibility within patient care areas [12,13]. Regular patient rounding, a proactive nursing practice involving scheduled checks on patients, is also recognized as a potential intervention to improve visibility and address patient needs preemptively, although its effectiveness in significantly reducing falls remains a subject of ongoing investigation [6,14-16].

While existing research provides valuable insights into how environmental design and workflow impact visibility and fall prevention, a significant gap persists: the need for objective and continuous visibility assessment in hospitals. Current methods, often relying on manual observation or simulation studies [12,17], are limited. Manual observation, while informative, is susceptible to observer bias and struggles to capture comprehensive spatial-temporal data over extended periods. For example, 1 study improving visibility used a labor-intensive approach with observers manually logging staff positions and actions at set intervals across designated zones [12]. Another study used simulations to calculate the number of beds visible from nursing stations and then relied on paired observers to track staff frequency in patient rooms and nurse stations. Room occupancy was logged each time staff entered, and stopwatches were used to measure staff time spent in different areas during 4-hour observation shifts [17]. Such methodologies, while providing snapshots of activity, may not fully represent the dynamic and nuanced nature of staff movement and the resulting visibility patterns within a hospital unit over time.

To address these limitations and advance the understanding of visibility in fall prevention, this research introduces the application of the real-time location system (RTLS). RTLS technologies offer an objective means to track the real-time location and movement of equipment within indoor environments [18,19]. Compared to manual observation, RTLS provides continuous, granular data on spatial occupancy and movement patterns, enabling a more comprehensive and data-driven analysis of visibility dynamics. Prior studies have demonstrated the utility of RTLS in health care for diverse applications, including space use analysis and workflow optimization [20,21]. However, the potential of RTLS to precisely track the movement trajectories of mobile resources, such as nursing carts (satellite nursing stations), and to conduct fine-grained spatial analysis of caregiver activity spaces in relation to patient falls remains largely unexplored.

The primary objective of this study is to leverage an RTLS to analyze the movement trajectories and spatial coverage of health care staff within hospital patient rooms and correlate these patterns with the incidence of hospital falls. Specifically, we hypothesize that the percentage of unvisited areas (spatial shadow zones) within patient rooms over a defined period may be positively associated with fall rates. Furthermore, this research aims to identify an optimal interval for regular staff rounding (eg, 60 minutes or another empirically derived timeframe) that balances proactive patient monitoring with efficient resource allocation. By quantifying the relationship between the temporal dynamics of staff visibility, the spatial distribution of unvisited areas, and fall occurrence, this study seeks to contribute to the development of evidence-based strategies for enhancing patient safety and optimizing staff workflow in hospital settings.

### Related Works

RTLS provides a means for continuously and in real-time determining the location of individuals or objects within indoor environments [18]. They have found increasing application within health care settings, primarily for the localization and tracking of medical equipment and personnel to enhance operational efficiency and optimize patient flow [19,22]. The selection of an appropriate RTLS technology is contingent upon the specific application requirements, with key considerations including positional accuracy, cost of deployment and maintenance, scalability, and potential for interference with medical devices [23]. Several RTLS technologies have been explored for health care applications, each with distinct characteristics. Radio-frequency identification (RFID) has been frequently investigated due to its relatively low cost; however, RFID systems typically offer room-level accuracy and are susceptible to interference with certain medical equipment [24]. Furthermore, achieving finer-grained location data with RFID often necessitates a denser infrastructure deployment, potentially increasing complexity and cost. Wireless Fidelity (Wi-Fi) based positioning leverages existing hospital infrastructure, as Wi-Fi networks are widely available in most health care facilities, thereby minimizing additional infrastructure investment. However, Wi-Fi positioning generally provides lower accuracy (in the range of 3-5 meters) and may require multiple access points to ensure adequate coverage and precision. Technologies such as ZigBee (Connectivity Standards Alliance) and Bluetooth Low Energy (Bluetooth Special Interest Group) offer advantages in terms of energy efficiency and tag miniaturization, but tend to exhibit lower positional accuracy compared to other modalities. Infrared systems can achieve high accuracy but are limited by line-of-sight requirements, restricting their applicability in complex and occluded hospital environments. Ultrasonic systems offer low cost and energy consumption but often suffer from poor scalability and challenges in tracking multiple moving objects concurrently [25].

In contrast to these alternatives, ultrawideband RTLS technology emerges as a particularly promising sensor choice for applications demanding high precision. Survey literature highlights ultrawideband’s capacity for centimeter-level accuracy, good resistance to multipath propagation, low power consumption, compact tag size, and high operating frequencies that mitigate interference [22]. While potentially incurring higher upfront costs compared to some other RTLS options, the superior accuracy and real-time tracking capabilities of ultrawideband are crucial for applications requiring detailed spatial analysis. For instance, while RFID-based RTLS may be suitable for asset tracking where room-level accuracy suffices, precise monitoring of staff workflows and movement trajectories necessitates the finer spatial resolution afforded by ultrawideband. A systematic review of RTLS technologies in health care also underscores ultrawideband as one of the most accurate technologies, capable of providing tracking precision down to 20 centimeters [22].

Given the objective of this study to investigate the association between mobile workstation movement patterns, spatial shadow zones within a health care unit, and the incidence of patient falls, the precise tracking of mobile workstation trajectories and the granular spatial analysis of caregiver activity spaces are paramount. Therefore, ultrawideband RTLS technology was selected for the technological implementation of this research. Its capacity to provide centimeter-level accuracy and real-time location updates makes it uniquely suited to capture the nuanced spatial dynamics of caregiver movement and to reliably quantify the percentage of spatial shadow zones. Furthermore, established methodologies for validating RTLS accuracy, often using cumulative distribution function (CDF) analysis of the distance error between reported and actual positions [25,26], provide a framework for assessing the performance of the deployed ultrawideband system, as detailed in the subsequent Methods section.

## Methods

### Study Design and Settings

This study was conducted within the acute care unit of a tertiary hospital, specifically within a medical observation area dedicated to patients who have been stabilized following emergency treatment but require ongoing care while awaiting inpatient ward admission. The patient population within this medical observation area primarily comprised acute medical patients who have been stabilized following initial emergency treatment but require continued monitoring and care while awaiting transfer to an inpatient ward. This population presented with a diverse range of diagnoses commonly encountered in emergency observation units, including, but not limited to, respiratory ailments, cardiovascular conditions, sepsis, and complications related to cancer.

This study’s setting comprised a 400-square-meter acute care unit characterized by a hybrid nurse station configuration. This configuration incorporated a central nursing station supplemented by mobile workstations to facilitate decentralized care delivery. These mobile workstations served as crucial nodes for clinical workflow, supporting a range of essential nursing tasks directly at the patient’s bedside. Functionally, mobile workstations were used for documenting patient information and reviewing patient histories, accessing supplies, managing waste disposal, facilitating real-time medication administration, and performing other immediate nursing care activities. Each mobile workstation weighs approximately 75 to 120 kg and is equipped with a computer, medical devices, and a work surface, as well as storage space for clinical supplies and medications [13,27]. Medical coverage within the unit was provided continuously by resident physicians in 2 shifts around the clock. Nursing care was delivered by registered nurses working in 3 shifts, with approximate patient-to-nurse ratios of 7:1 during daytime shifts, 9:1 during evening shifts, and 10:1 during nighttime shifts. Data collection for both the RTLS and patient outcomes spanned a period of 210 days, from August 4, 2024, to March 1, 2025, encompassing a total of 30 weeks.

### RTLS Infrastructure

**Figure 1:**
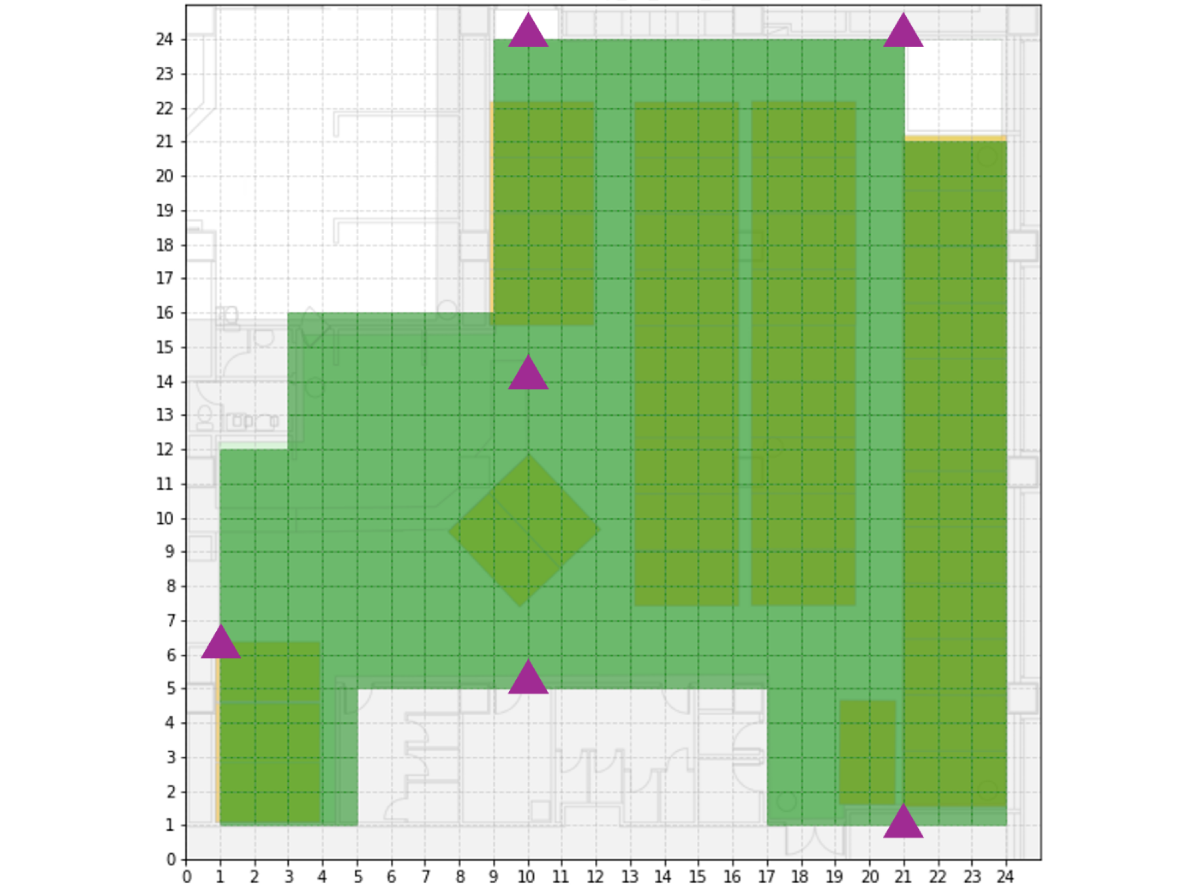
Acute care unit layout and RTLS anchor placement. Depiction of the acute care unit study setting. The unit area, delineated in green, is virtually partitioned into a grid of 1-meter by 1-meter cells. Purple triangles indicate the locations of the 6 ultrawideband RTLS anchors deployed within the unit. Anchor placement was constrained by existing power outlet locations and medical building infrastructure regulations. RTLS: real-time location system.

An ultrawideband RTLS was deployed within the acute care unit to continuously monitor the location of mobile workstations (Figure 1). The RTLS infrastructure consisted of 6 ultrawideband anchors strategically positioned at fixed locations throughout the unit to ensure comprehensive spatial coverage. Ultrawideband tags were affixed to each mobile workstation. These tags emitted radio signals at a frequency of 1 Hz during periods of motion. To optimize battery life, the tag transmission rate was reduced to 1 signal per minute when the mobile workstation remained stationary. The ultrawideband anchors received signals from the tags, and this data was transmitted to a central server. The server used time-of-flight algorithms to calculate the 3D coordinates of each mobile workstation within the unit in real time.

### Outcomes

The primary outcome measure for this study was the incidence of hospital falls within the acute care unit. Hospital falls were defined as unintentional events resulting in a person coming to rest on the ground. Data on fall events were extracted from the hospital’s incident reporting system. The secondary outcome measure was patient clinical deterioration requiring transfer to the intensive care unit (ICU). Data on these transfers were obtained from hospital electronic health records.

### Spatial Data Processing and Analysis

To facilitate spatial analysis, the 400 square meter acute care unit was virtually partitioned into a grid of 1-meter by 1-meter square cells. For each mobile workstation, a “coverage area” was defined based on its real-time location. This coverage area was operationalized as the grid cell occupied by the mobile workstation, expanded by a spatial dilation of 2 grid cells in all cardinal and diagonal directions. This spatial dilation was implemented to approximate the visual and functional range of a caregiver in proximity to the mobile workstation. A dilation distance of 2 meters was selected, which is consistent with established literature defining the personal and social zones in proxemics theory and interpersonal distances [28,29]. Informed by Hall [29] distance theory, particularly the characteristics of the social and personal zones, this study operationalizes caregiver visibility by defining a coverage area around mobile workstations based on a spatial dilation of 2 meters. This 2-meter radius is intended to represent an approximate zone within which caregivers, when positioned with a mobile workstation, can reasonably be expected to maintain visual awareness and effectively respond to patient needs, balancing the need for proximity with practical considerations of workflow and spatial constraints in the acute care setting. We recognize that actual caregiver visibility and responsiveness in a dynamic acute care setting are influenced by a multitude of factors, including line-of-sight obstructions, physical barriers, and variability in clinical tasks. Thus, this 2-meter radius serves as a standardized operational definition for quantifying spatial coverage for the purposes of this specific research study. To assess the findings of this specific operationalization, sensitivity analyses were systematically conducted across a range of spatial dilation distances (1 to 4 meters), as detailed in the Sensitivity Analyses section.

### RTLS Accuracy Validation

Before data collection, a validation procedure was conducted to assess the positional accuracy of the deployed RTLS within the acute care unit. A predefined route, representative of typical mobile workstation movement patterns within the unit, was established and marked. Four ultrawideband tags, affixed to a mobile workstation, were then moved along this designated route at a controlled speed of approximately 0.5 to 1 meter per second. Simultaneously, location data was recorded by the RTLS. To quantify positional accuracy, the error distance for each recorded RTLS position was calculated by comparing it to the corresponding ground truth position along the predefined route. The distribution of these error distances was then visualized using a CDF plot.

### Definition of Spatial Shadow Zones and Percentage of Spatial Shadow Zone

Spatial shadow zones were defined as grid cells within the acute care unit that were not included within the coverage area of any mobile workstation for a continuous period of 60 minutes. This 60-minute interval was selected as consistent with previous literature on rounding frequency [6,14]. The percentage of spatial shadow zone was calculated as the total area of spatial shadow zones within the unit, divided by the total area of the acute care unit (400 square meters). This percentage was calculated for each minute to represent the temporal dynamics of unvisited areas.

### Sensitivity Analyses

To assess the robustness of the primary findings, sensitivity analyses were conducted by systematically varying key parameters in the spatial analysis. First, the spatial dilation distance defining the mobile workstation coverage area was varied across a range of 1 to 4 meters (1-meter, 2-meter, 3-meter, and 4-meter dilation distances were tested). Second, the temporal threshold for defining spatial shadow zones was examined across multiple durations, ranging from 15 to 90 minutes (specifically, periods of 15, 30, 45, 60, 75, and 90 minutes were evaluated). These variations were implemented to explore the sensitivity of the results to different operationalizations of caregiver visibility and unvisited areas.

In addition to parameter variations, a temporal sensitivity analysis was performed by dividing the total 210-day study period into 2 equal segments: the first 15 weeks and the subsequent 15 weeks. The primary analyses were then repeated separately for each of the 15 weeks. This temporal segmentation was conducted to investigate potential variations in the observed relationships over time, and to assess the consistency of the findings across different phases of the data collection period.

### Statistical Analysis

Data processing and spatial analyses were conducted using Python (version 3.11.9; Python Software Foundation). Statistical analyses were performed using the R statistical software environment (version 4.3.1; R Foundation). Continuous variables were compared between independent groups using the independent samples *t* test (2-tailed). To assess the association between the percentage of spatial shadow zone and the dichotomous outcomes of hospital falls and ICU transfer, multivariable logistic regression analysis was used. In these regression models, the percentage of spatial shadow zone served as the primary independent variable. To account for potential confounding effects related to temporal variations in hospital activity and staffing, we adjusted for day of the week and patient-to-nurse ratio as covariates in the multivariable models. All statistical tests were 2-tailed, and statistical significance was defined as a *P* value less than .05.

### Ethical Considerations

This study was granted an exemption from full review by the National Taiwan University Hospital Research Ethics Committee (NTUH-REC number: 202403045W; March 28, 2024). As this study involved retrospective analysis of anonymized, aggregated location data and did not involve direct interaction with human subjects, informed consent was not required and was not obtained. The study utilized aggregated, anonymized data, and no patient or staff identifying information was collected or analyzed, to make the data collected from the systems of that specific place as discrete as possible. Given the retrospective observational nature of the study and the lack of direct interaction with participants, no compensation was provided for participation.

## Results

### Study Population and Event Incidence

Over the 210-day study period, a total of 97 adverse events were recorded within the acute care unit. Among these, 8 were identified as hospital falls. Given the unit’s 40-bed capacity and the 210-day observation period, the hospital fall rate was calculated as 1.0 per 1000 patient-days. Additionally, 89 patients experienced clinical deterioration necessitating transfer to the ICU.

Analysis of faller type revealed that 6 falls involved patients, 1 involved a patient’s family member, and 1 involved a staff member. The temporal distribution of fall events indicated occurrences across various hours of the day: 7 AM (n=2), 9 AM (n=1), 10 AM (n=1), 11 AM (n=1), 4 PM (n=1), and 11 PM (n=2). The distribution of causes for ICU transfer was as follows: advanced respiratory support (n=45), circulatory support (n=35), cardiac arrest (n=4), neurological monitoring (n=3), acute coronary syndrome (n=1), and diabetic ketoacidosis (n=1). The temporal distribution of ICU transfers based on the nursing shift structure was: nighttime shift (n=16), daytime shift (n=24), and evening shift (n=49; Multimedia Appendices 1 and 2).

### RTLS Positional Accuracy

**Figure 2:**
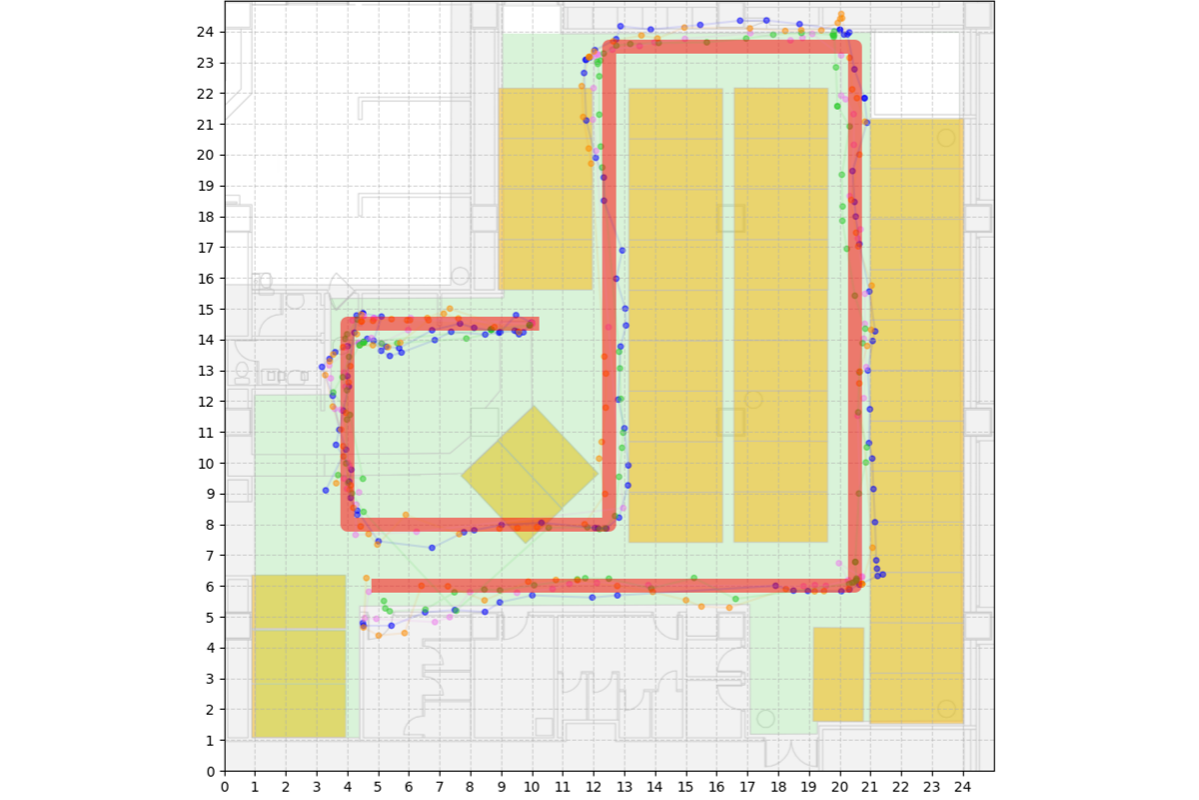
RTLS positional accuracy validation trajectory. Visualization of the RTLS positional accuracy validation procedure. The red line delineates the predefined ground truth route, representing a typical mobile workstation movement trajectory within the acute care unit. Colored dots represent the 419 positional estimates recorded by the RTLS from 4 ultrawideband tags affixed to a mobile workstation as it was moved along the predefined route. Yellow rectangles indicate the locations of the 40 patient beds within the unit. RTLS: real-time location system.

**Figure 3:**
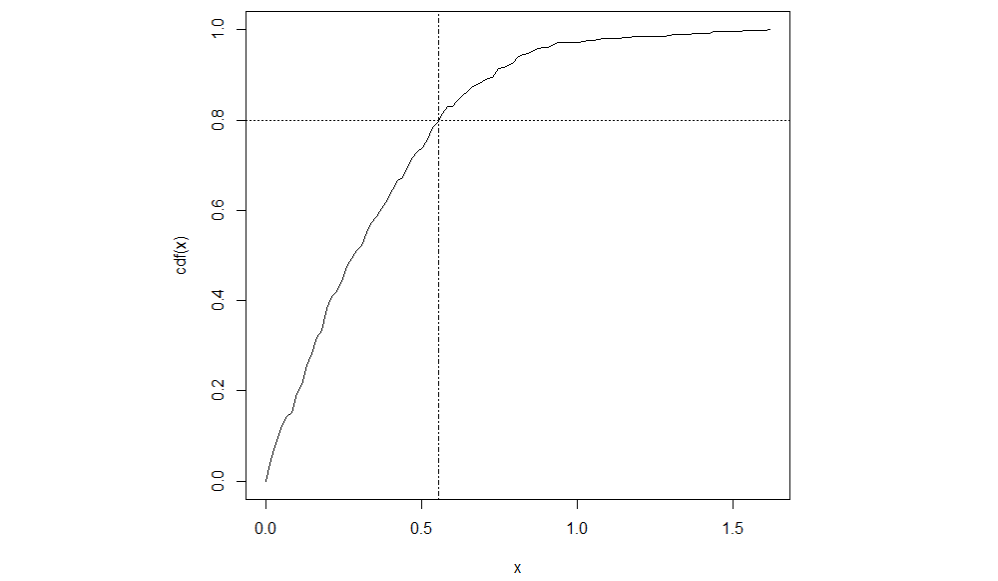
CDF of RTLS positional error distances. CDF plot illustrating the distribution of positional error distances from the RTLS accuracy validation. The x-axis represents the positional error distance in meters, and the y-axis represents the cumulative probability. The vertical dashed line at x=0.554 meters and the horizontal dashed line at y=0.8 indicate that 80% of the RTLS positional estimates were within an error distance of 0.554 meters or less. CDF: cumulative distribution function; RTLS: real-time location system.

To validate the accuracy of the RTLS, a dedicated accuracy assessment was performed. Data from 4 ultrawideband tags, collected along a predefined route, yielded 419 positional estimates (Figure 2). Descriptive statistics for the positional error, defined as the distance between the RTLS-estimated positions and the ground truth route, revealed a mean error of 0.346 meters, 95% CI 0.319 to 0.373. The CDF of positional errors is provided in Figure 3, visually illustrating the distribution of RTLS accuracy.

### RTLS Data Quality Metrics

Analysis of RTLS data quality revealed a mean missing data rate of 0.376, 95% CI 0.369 to 0.383 per hour, after excluding periods of ultrawideband tag inactivity-induced data reduction. Furthermore, an evaluation of potential data artifacts identified instances of spurious positional displacement, characterized as isolated outlier points relative to preceding and subsequent positions more than 2.5 meters. Across a total of 47,230,357 recorded location data points, 106,564 points were classified as noise according to these criteria, representing an overall noise rate of approximately 0.226% (1 in 443 data points).

### Spatial Shadow Zone and Clinical Outcomes

Descriptive statistics for the percentage of spatial shadow zone, stratified by fall events and ICU transfers, are presented in Table 1. The mean percentage of spatial shadow zone was numerically higher in instances of falls, exhibiting an average increase of 1.0 percentage point (95% CI 0.0 to 1.9), compared to nonfall events. When the analysis was restricted to patient falls only (excluding falls involving staff and visitors), the difference in mean percentage of spatial shadow zone remained numerically higher in fall events but was no longer statistically significant, demonstrating an average increase of 0.4 percentage points (95% CI −0.7 to 1.5). Conversely, the mean percentage of spatial shadow zone was statistically significantly lower in instances of ICU transfer, demonstrating an average decrease of 1.1 percentage points (95% CI 0.8 to 1.4), when compared to non-ICU transfer events. Independent samples *t* tests indicated a statistically significant difference in the mean percentage of spatial shadow zone between fall and nonfall events (*P*=.04) and between ICU transfer and non-ICU transfer events (*P*<.001).

**Table 1:** Percentage of spatial shadow zone by event occurrence.

Event	Percentage of spatial shadow zone (%), mean (SD)	*P* value
Overall (n=250,363)	76.8 (10.3)	—^a^
Fall events (n=470)	77.8 (10.9)	.04
Nonfall events (n=249,893)	76.8 (10.3)	.04
ICU^b^ transfer events (n=4659)	75.8 (10.0)	<.001
Non-ICU transfer events (n=245,704)	76.9 (10.3)	<.001

^a^Not applicable.

^b^ICU: intensive care unit.

### Multivariable Logistic Regression Analysis of Hospital Falls

The results of the multivariable logistic regression analysis examining the association between the percentage of spatial shadow zone and the odds of hospital falls are presented in Table 2. After adjusting for patient-to-nurse ratio and day of the week, the percentage of spatial shadow zone was significantly associated with an increased odds of hospital falls (odds ratio 1.02, 95% CI 1.01 to 1.03, *P*<.001). The coefficients for the day-of-week were not statistically significant (*P*>.05).

**Table 2:** Multivariable logistic regression analysis of hospital falls.

Predictor	Odds ratio (95% CI)	*P* value
Percentage of spatial shadow zone	1.02 (1.01 to 1.03)	.001
Patient-to-nurse ratio	0.84 (0.78 to 0.90)	<.001
Day of week (adjusted)	—^a^	>.05

^a^Not applicable.

### Sensitivity Analyses Results

To assess the robustness of the primary finding regarding the association between the percentage of spatial shadow zone and hospital falls, sensitivity analyses were conducted by varying the spatial dilation distance and the temporal threshold for defining spatial shadow zones. Independent samples *t* tests were performed to compare the mean percentage of spatial shadow zone between fall and nonfall events across these varied parameters.

As shown in Table 3, the statistical significance of the difference in mean percentage of spatial shadow zone between fall and nonfall events varied depending on the spatial dilation distance and the temporal threshold for shadow zone definition. While the effect size was dependent on the choice of these parameters, statistically significant differences were observed across several parameter combinations. When the spatial dilation distance was reduced to 1 meter, statistically significant differences were observed across temporal thresholds ranging from 15 to 75 minutes. However, as the dilation distance increased, statistical significance was attenuated and lost at temporal thresholds of 60 minutes and above. With a dilation distance of 2 meters (the primary analysis parameter), statistical significance was observed at temporal thresholds of 15, 30, 45, and 60 minutes, but not at 75 or 90 minutes.

These sensitivity analyses suggest that the observed association between the percentage of spatial shadow zone and hospital falls was most robust within a temporal shadow zone threshold of up to 45 minutes. Beyond these parameters, the statistical evidence for a significant association weakens.

In addition to varying spatial and temporal parameters for shadow zone definition, a temporal sensitivity analysis was conducted to assess the consistency of findings across different phases of this study’s period. In the analysis of the first 15-week period, the mean percentage of spatial shadow zone was numerically higher for fall events (mean 78.5) compared to nonfall events (mean 77.6), but this difference was not statistically significant (*P*=.15, 95% CI −0.3 to 2.2). Similarly, for the subsequent 15-week period, while the mean percentage of spatial shadow zone remained numerically higher for fall events (mean 77.0) compared to nonfall events (mean 76.0), this difference also did not reach statistical significance (*P*=.14, 95% CI −0.3 to 2.4).

**Table 3:** Sensitivity analysis and 95% CIs for the difference in means of percentage of spatial shadow zone between nonfall and fall events across varying spatial dilation distances and temporal shadow zone thresholds.

Temporal shadow zone thresholds (minutes)	Dilation distance (meters), 95% CI			
	1	2	3	4
15	1.38 to 3.08^a^	2.67 to 6.27^a^	3.81 to 9.48^a^	4.45 to 11.47^a^
30	0.98 to 2.20^a^	1.54 to 4.12^a^	2.00 to 6.07^a^	2.40 to 7.43^a^
45	0.42 to 1.43^a^	0.51 to 2.65^a^	0.60 to 3.98^a^	0.79 to 4.97^a^
60	0.24 to 1.12^a^	0.03 to 1.90^a^	−0.17 to 2.78	−0.13 to 3.51
75	0.07 to 0.86^a^	−0.34 to 1.34	−0.82 to 1.83	−1.02 to 2.26
90	−0.07 to 0.66	−0.76 to 0.79	−1.49 to 0.96	−1.87 to 1.15

^a^Statistically significant.

## Discussion

### Principal Results

This study provides novel evidence for a significant positive association between the percentage of spatial shadow zone within an acute care unit and the incidence of hospital falls. Leveraging an RTLS for objective and continuous measurement of mobile workstation spatial coverage, we found that a higher percentage of unvisited areas over time was associated with substantially increased odds of fall events. This finding underscores the critical role of caregiver visibility and proactive patient monitoring in mitigating fall risk within hospital settings. Conversely, we observed an inverse relationship between the percentage of spatial shadow zone and the odds of ICU transfer.

The observed positive association between spatial shadow zones and hospital falls is congruent with the established understanding of visibility as a crucial extrinsic factor in patient safety and fall prevention [10]. Spatial shadow zones, as operationally defined in this study, represent periods and locations within the patient care environment where mobile workstations—and by extension, readily available caregivers—were absent for a continuous 60-minute interval. These extended periods of reduced caregiver presence could diminish opportunities for routine patient monitoring, proactive identification of evolving patient needs, and timely intervention to prevent potential falls. Our findings build upon and extend prior research that has emphasized the importance of unit layout design [17], nursing station configuration [12], and structured rounding practices [16] in shaping caregiver visibility and influencing patient outcomes. The odds ratio observed in our analysis suggests that interventions aimed at reducing spatial shadow zones might represent a potent strategy for enhancing fall prevention efforts.

The observed inverse relationship between spatial shadow zone and ICU transfers necessitates interpretation within the clinical context of this acute care unit. Specifically designed for patients in a transitional phase of postemergency stabilization, yet with preinpatient ward admission, this unit manages a patient population characterized by inherently dynamic and potentially labile conditions. While proactive rounding and consistent caregiver presence are generally posited to enhance patient monitoring, thereby potentially mitigating adverse events, they may not directly avert all instances of clinical deterioration requiring ICU escalation. Our interpretation is that in the temporal window preceding ICU transfer events, the exigency of deteriorating patient conditions could have likely prompted heightened nursing vigilance and intensified bedside care delivery. This increased demand for immediate, patient-centered interventions was typically observed to result in sustained caregiver presence at the patient’s bedside, with mobile workstations consequently deployed and used near acutely ill individuals. This focused allocation of nursing resources and bedside workstation use would, in turn, reduce the percentage of spatial shadow zone within the unit during the 60-minute interval preceding ICU transfer. Under this interpretation, the diminished spatial shadow zone is not a protective factor preventing ICU transfer, but rather a potential marker of escalating patient acuity and the resultant, appropriately intensified nursing response characterized by concentrated bedside care.

### Comparison With Prior Work

Several prior studies have investigated the impact of hourly rounding on patient fall rates, often using cohort study designs to analyze pre- and postimplementation fall rates [14-16]. These studies generally hypothesize that hourly rounding, by increasing caregiver presence and proactive patient interaction, would reduce the occurrence of spatial shadow zones within patient units. Our sensitivity analyses, exploring varying temporal thresholds for shadow zone definition, suggest that a 45-minute interval may represent a more robust and potentially clinically relevant timeframe for observing significant associations with fall risk, particularly when considering the spatial dilation of mobile workstation coverage areas. While some research suggests that increasing rounding frequency beyond hourly intervals (eg, every 30 minutes or more frequently) does not necessarily yield further reductions in fall rates [16] and may indeed increase caregiver workload, our study focuses on the percentage of spatial shadow zone as a continuous metric rather than discrete rounding intervals. This approach offers a nuanced perspective on the temporal dynamics of caregiver visibility beyond simply the frequency of scheduled rounds.

Hospital fall rates, typically reported in the range of 2 to 8 falls per 1000 patient-days [8], are recognized as a relatively infrequent patient safety indicator, posing challenges for detecting statistically significant associations with interventions or risk factors, particularly in studies with limited sample sizes or observation periods [14]. Our temporal sensitivity analysis, segmenting the 210-day study period, indicated a consistent trend of numerically higher shadow zone percentages preceding fall events across both time segments, although statistical significance was not consistently achieved in these segmented analyses. This may reflect the inherent variability in fall occurrence and the need for larger datasets or longer observation periods to detect temporal relationships, especially for less frequent events.

The implementation of RTLS technology for objective spatial measurement represents a key methodological advancement in this study. Compared to prior work relying on manual observation or proxy measures of visibility, RTLS enables continuous and granular data collection, overcoming limitations inherent in traditional methods [12,17]. Our RTLS positional accuracy, with a mean error of 35 cm and approximately 70% of measurements within 45 cm of the ground truth, is slightly worse than previously reported accuracy for ultrawideband systems in ideal spatial settings, such as the 25 cm mean error and 70% within 40 cm reported by Silvia et al [25].

### Limitations

There are several limitations in this study. This study was conducted within a single acute care unit in a tertiary hospital, potentially limiting the generalizability of our findings to other hospital settings with different patient populations, unit layouts, staffing models, or care protocols. The operational definition of spatial shadow zone, while informed by clinical considerations and practical feasibility, remains a specific metric, and future research could explore alternative definitions, temporal thresholds, and spatial parameters. Furthermore, while multivariable regression models adjusted for temporal factors such as day of the week, residual confounding by unmeasured or imperfectly measured variables cannot be entirely excluded. In particular, this analysis lacked granular data on individual-level characteristics, such as patient acuity, nurse experience, or visitor or staff health status, which may potentially affect the results. Finally, our outcome measures relied on hospital incident reporting systems and electronic health record data, which may be subject to potential underreporting, misclassification, or inconsistencies in data capture. This study does not include a power calculation or formal sample size justification for the number of observed fall events, given the relative rarity of this outcome, and raises concerns about the statistical power to detect meaningful associations.

Despite these limitations, this study provides evidence for the association between the percentage of spatial shadow zones and hospital falls. Moving forward, these findings have several implications for patient safety and health care operations. The quantifiable relationship between spatial shadow zones and hospital falls suggests that proactively minimizing unvisited areas within acute care units might be a valuable and targeted strategy for fall prevention. Hospital design and unit layout should prioritize maximizing caregiver visibility and minimizing spatial configurations that contribute to prolonged periods of reduced surveillance. Real-time monitoring of spatial shadow zones, enabled by RTLS technology, could serve as a proactive risk indicator, prompting targeted interventions during periods or in areas with elevated shadow zone percentages. Future research should focus on interventional studies to directly test the impact of strategies specifically aimed at reducing spatial shadow zones on hospital fall rates and other relevant patient outcomes. Multicenter studies encompassing diverse hospital settings and patient populations are needed to enhance the generalizability of these findings. Investigation into the inverse association with ICU transfers is crucial to elucidate its underlying mechanisms and clinical significance, potentially informing more nuanced and context-specific strategies for optimizing caregiver allocation and patient monitoring in acute care environments. Subsequent research should also explore the potential for leveraging RTLS technology to develop intelligent, real-time decision support systems, perhaps akin to a “fog of war” visualization, to proactively guide caregiver rounding and resource allocation, aiming to systematically and efficiently maintain low spatial shadow zone percentages without increasing caregiver workload. Future research should also address what can improve clinician efficiencies, decrease burnout, potentially serve as an early warning system for changes in patient acuity, and be integrated into existing health care environments.

### Conclusions

Leveraging RTLS technology, this research objectively quantified spatial shadow zones and demonstrated their potential as a novel, quantifiable risk indicator associated with hospital falls. The findings underscore the importance of hospital design and workflow optimization strategies that prioritize maximizing caregiver spatial coverage and minimizing prolonged periods of unvisited patient areas, while giving a quantifiable metric tool that can assist in how the areas are more precisely monitored.

## Appendix

Multimedia Appendix 1 Dynamic visualization of spatial shadow zones: fog of war analogy. This video illustrates the minute-by-minute evolution of spatial shadow zones over a 1-hour period. Areas in proximity to mobile workstations are visually illuminated, while shadow zones, representing areas unvisited for the defined temporal threshold, progressively darken, analogous to the "fog of war" concept in real-time strategy games.

Multimedia Appendix 2 Real-time dynamics of spatial shadow zones: a "fog of war" representation. This video demonstrates the temporal dynamics of spatial shadow zones within the acute care unit over a 60-minute period. Areas within the coverage radius of mobile workstations are depicted as "illuminated," while regions exceeding the temporal shadow zone threshold become progressively darker, visually mimicking the receding "fog of war" as caregiver presence wanes.

## Abbreviations

CDF: cumulative distribution function
ICU: intensive care unit
RFID: radio-frequency identification
RTLS: real-time location system
Wi-Fi: Wireless Fidelity
